# Cardiogenic shock and acute kidney injury: the rule rather than the exception

**DOI:** 10.1007/s10741-020-10034-0

**Published:** 2020-10-02

**Authors:** N Ghionzoli, C Sciaccaluga, GE Mandoli, G Vergaro, F Gentile, F D’Ascenzi, S Mondillo, M Emdin, S Valente, M Cameli

**Affiliations:** 1 Department of Medical Biotechnologies, Division of Cardiology, University of Siena, Policlinico Le Scotte, Viale Bracci 16, Siena, Italy; 2 Institute of Life Sciences, Scuola Superiore Sant’Anna, Pisa, Italy; 3 Division of Cardiology and Cardiovascular Medicine, Fondazione Toscana Gabriele Monasterio, Pisa, Italy

**Keywords:** Cardiogenic shock, Acute kidney injury, Heart failure, Replacement therapy, Outcome

## Abstract

Cardiogenic shock (CS) is a life-threatening condition of poor end-organ perfusion, caused by any cardiovascular disease resulting in a severe depression of cardiac output. Despite recent advances in replacement therapies, the outcome of CS is still poor, and its management depends more on empirical decisions rather than on evidence-based strategies. By its side, acute kidney injury (AKI) is a frequent complication of CS, resulting in the onset of a cardiorenal syndrome. The combination of CS with AKI depicts a worse clinical scenario and holds a worse prognosis. Many factors can lead to acute renal impairment in the setting of CS, either for natural disease progression or for iatrogenic causes. This review aims at collecting the current evidence-based acknowledgments in epidemiology, pathophysiology, clinical features, diagnosis, and management of CS with AKI. We also attempted to highlight the major gaps in evidence as well as to point out possible strategies to improve the outcome.

## Introduction

Cardiogenic shock (CS) is a critical condition of end-organ hypoperfusion, consequent to a severe decrease in cardiac output, in spite of adequate intravascular volume. Therefore, hypotension requiring volume resuscitation and signs of end-organ hypoperfusion represent the clinical landmarks of CS, urgently demanding for pharmacological and/or mechanical intervention [1–3].

Acute kidney injury (AKI) represents a sudden insult to renal function that encompasses several clinical scenarios, ranging from a mild increase in serum creatinine to end-stage renal disease, as stated by Risk-Injury-Failure-Loss-End-stage (RIFLE) criteria. In order to make a diagnosis of AKI, at least one of the following criteria has to be met: increase in serum creatinine ≥ 0.3 mg/dL within 48 h; increase in basal serum creatinine by ≥ 1.5 times within the previous 7 days; urine volume < 0.5 mL/kg/h for 6 h [4]. For further details about RIFLE criteria, please see Table 1.

**Table 1:** Staging of acute kidney injury according to Kidney Disease: Improving Global Outcomes (KDIGO). Five stages of renal impairment have been described and ranked in ascending order according to the severity

Stage	Serum creatinine	Glomerular filtration rate	Urine output (mL/kg)
R: risk	1.5-fold increase	25% decrease	< 0.5 in 6 h
I: injury	2-fold increase	50% decrease	< 0.5 in 12 h
F: failure	3-fold increase or value ≥ 4 mg/dL	75% decrease	< 0.3 in 24 h (oliguria) or anuria for 12 h
L: loss (of function)	Complete loss of renal function for ≥ 4 weeks, requiring dialysis		
E: end stage	Uremia or complete loss of renal function for ≥ 3 months, requiring dialysis		

Management of both CS and AKI is strictly time-dependent: the longer they persist, the higher is the likelihood of developing irreversible organ damages. They can occur alone as well as clustered in the same patients. Evidences of inter-dependency between the heart and the kidney are described as cardiorenal syndromes (CRSs) [5]. With concerns to the specific setting of critical care, the most prevalent is type-1 CRS, whose pivotal pathogenetic mechanism lays on an abrupt decrease in renal perfusion, typical of patients with CS. Since kidneys are considered end-organs, it follows that a setting of CS can be the cause itself of AKI [1, 6]. Type-2 CRS refers to patients with a chronic heart disease that progressively affects renal function, e.g., chronic heart failure; type-3 CRS refers to patients with an abrupt reduction in renal function, thus leading to an acute cardiac disorder, i.e., glomerulonephritis causing fluid retention, hypertension, and then heart failure; type-4 CRS regards the consequences of chronic kidney disease on the cardiovascular system, i.e., vascular calcification; type 5 is for all the systemic disorders that affect both cardiac and renal functions, i.e., diabetes mellitus [6].

Nevertheless, whether many steps have been taken towards the comprehension of the mechanisms underlying both the conditions, few improvements have been made regarding effective therapies for this rare but often fatal disease. Still, a prompt intervention represents the leading modifier of the outcome for these patients.

## Epidemiology

Data regarding epidemiological features of CS combined to AKI are still poor. Most of them derive from patients with acute myocardial infarction (AMI), as it represents the leading cause of CS [7]. Hence, the applicability of the available data may be nebulous in clinical settings other than AMI. Thus, further studies investigating CS-AKI when other etiologies are responsible for CS should be promoted.

The incidence of AKI complicating CS (type-1 CRS) is considerably high, since it ranges from 20 to 35% according to studies [8]. This cohort was also burdened by a higher rate of complications, in-hospital mortality, and healthcare sources utilization as compared with patients suffering from only CS. Furthermore, the more impaired renal function is, the higher is the mortality, so that patients requiring hemodialysis had worse outcome than those who did not need it [9]. In a Danish population of 5079 patients with CS, 13% developed AKI requiring renal replacement therapy (RRT). Among them, the in-hospital mortality was 62% for those who required RRT and 36% for those who did not; this trend was further confirmed on a 5-year follow-up analysis, with a mortality of 43% for the first group and 29% for the second one [10]. In a US population of 440,257 patients admitted for CS complicating AMI, 35.3% developed AKI, and 3.4% AKI requiring hemodialysis. All-cause in-hospital mortality was higher in CS-AKI patients than in those with only CS, with a poorer trend in those who needed hemodialysis. Additionally, length of stay was proportionally higher in patients with CS without AKI, with AKI, and with AKI requiring RRT (9 ± 10, 12 ± 13, and 18 ± 19 days, respectively, *p* < 0.001) [11]. As a confirmation, AKI was often found to be an independent predictor of mortality in CS [12, 13].

Of note, gender analyses have shown uneven results. Males suffered from CS-AKI significantly more than females, as well as from other end-organs failure, despite women were older and with more comorbidities at presentation [14, 15]. On the other hand, in-hospital mortality was found to be surprisingly higher in women. The lower rate of coronary angiography performed in women as compared with men may at least partially explain this divergence, since prompt revascularization was not always guaranteed [6]. In contrast, gender did not influence in-hospital mortality in a setting of post-cardiotomy CS complicated by AKI [16].

## Pathophysiology

All cardiorenal syndromes are usually described as the mixing of either hemodynamic or non-hemodynamic factors, with type 1 making no exception [6]. The abrupt reduction in renal perfusion due to pump failure reduces the ability of the nephron to filter, with consequent reduction in urine output. This setting is described as prerenal AKI, since the leading cause is upstream to the kidney itself.

Moreover, especially when RV failure occurs, an increase in central venous pressure (CVP) may be observed, with consequent renal venous congestion and so loss of function [17]. The clinical relevance of such mechanism was further confirmed by a recent study by van den Akker et al., as they found that CVP was the only independent predictor for AKI in the context of CS, and by the role of RV performance in this particular clinical scenario [18, 19].

**Fig. 1:**
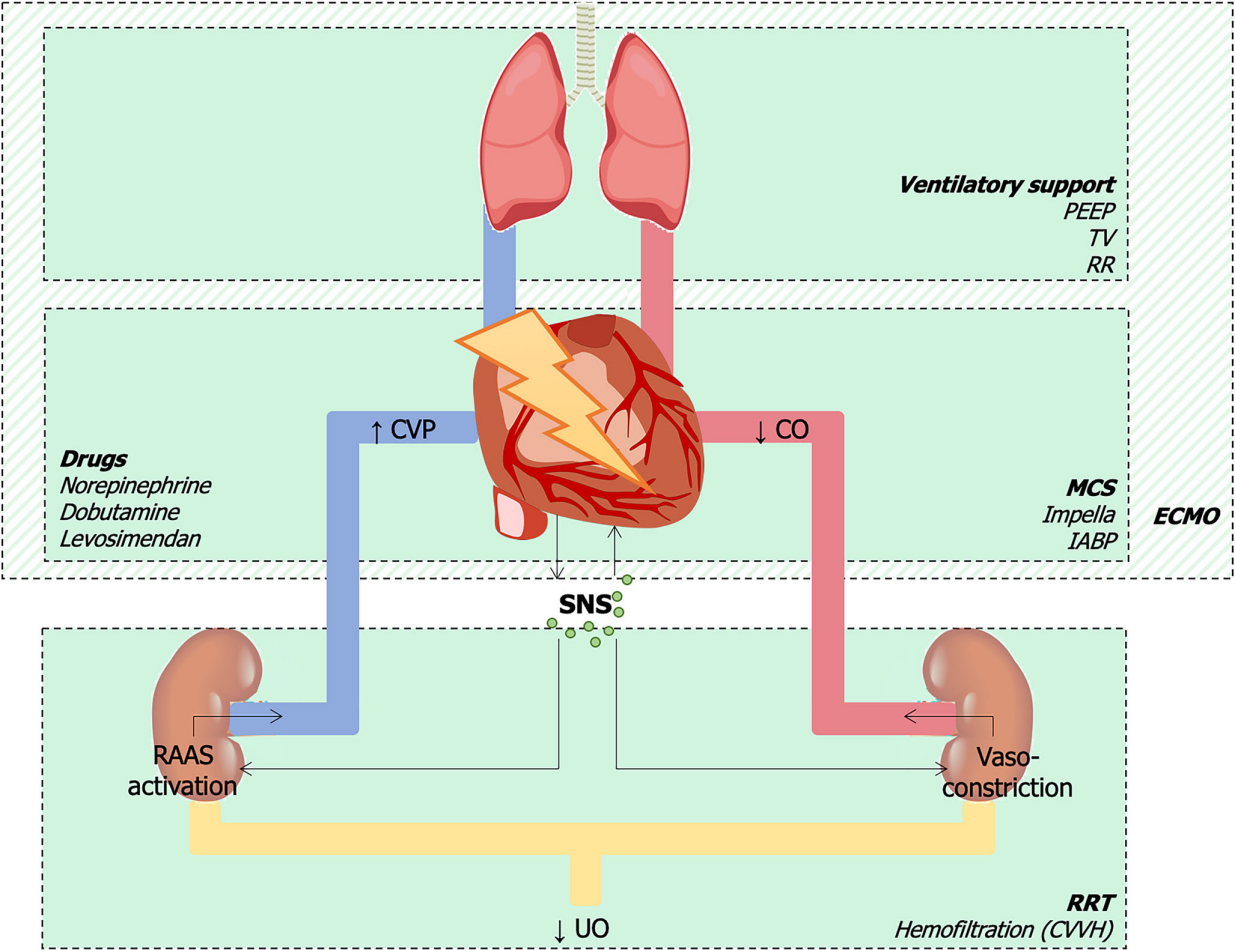
Hemodynamic and non-hemodynamic factors in CS, their interplay with the kidneys, and associated therapeutic strategies. Injuries to the heart can determine both a reduction in cardiac output and an increase in central venous pressure. Alongside, the activation of the sympathetic nervous system induces renal vasoconstriction and RAAS activation, thus reducing urine output. Replacement and pharmacological strategies are displayed for each organ. *CO*, cardiac output; *CVP*, central venous pressure; *CVVH*, continuous veno-venous hemofiltration; *IABP*, intra-aortic balloon pump; *MCS*, mechanical circulatory support; *PEEP*, positive end-expiration pressure; *RAAS*, renin-angiotensin-aldosterone system; *RR*, respiratory rate; *RRT*, renal replacement therapy; *SNS*, sympathetic nervous system; *TV*, tidal volume; *UO*, urinary output

Beyond these hemodynamic factors, the acute reduction in heart function is the *primum movens* for the activation of several neurohormonal systems, aiming at restoring hemodynamic stability. The balance of these molecular determinants is responsible for both the activation of life-saving pathways and detrimental effects. In this regard, the massive activation of the adrenergic system in CS has been well established, with proven positive inotropic effect and peripheral vasoconstriction [20, 21]. Although this response is partially adaptive in supporting vital functions, vasoconstriction involves also the kidneys. This leads to possible ischemic effects when autoregulation is exceeded—especially at the renal medulla—and is overall responsible for an increase in cardiac afterload [22, 23]. Furthermore, beta-1 stimulation of the kidney induces renin release, thus activating the renin-angiotensin-aldosterone cascade. The consequent sodium-water retention further increases cardiac afterload (Fig. 1). Whether the stimulation of mineralocorticoid receptors in the acute setting may be harmful still has to be clarified, especially with regard to non-epithelial tissues such as the heart.

The double-edged nature of neurohormonal drivers is further confirmed by the current clinical use of inotropes in the setting of CS. Norepinephrine and dobutamine are the most used agents, but their long-standing use is associated with an increased risk of cardiac arrhythmias, as well as severe ischemia in multiple organs [24]. Safer approaches may derive from replacement therapies, including both ultrafiltration and cardiac devices for transient mechanical support.

**Fig. 2:**
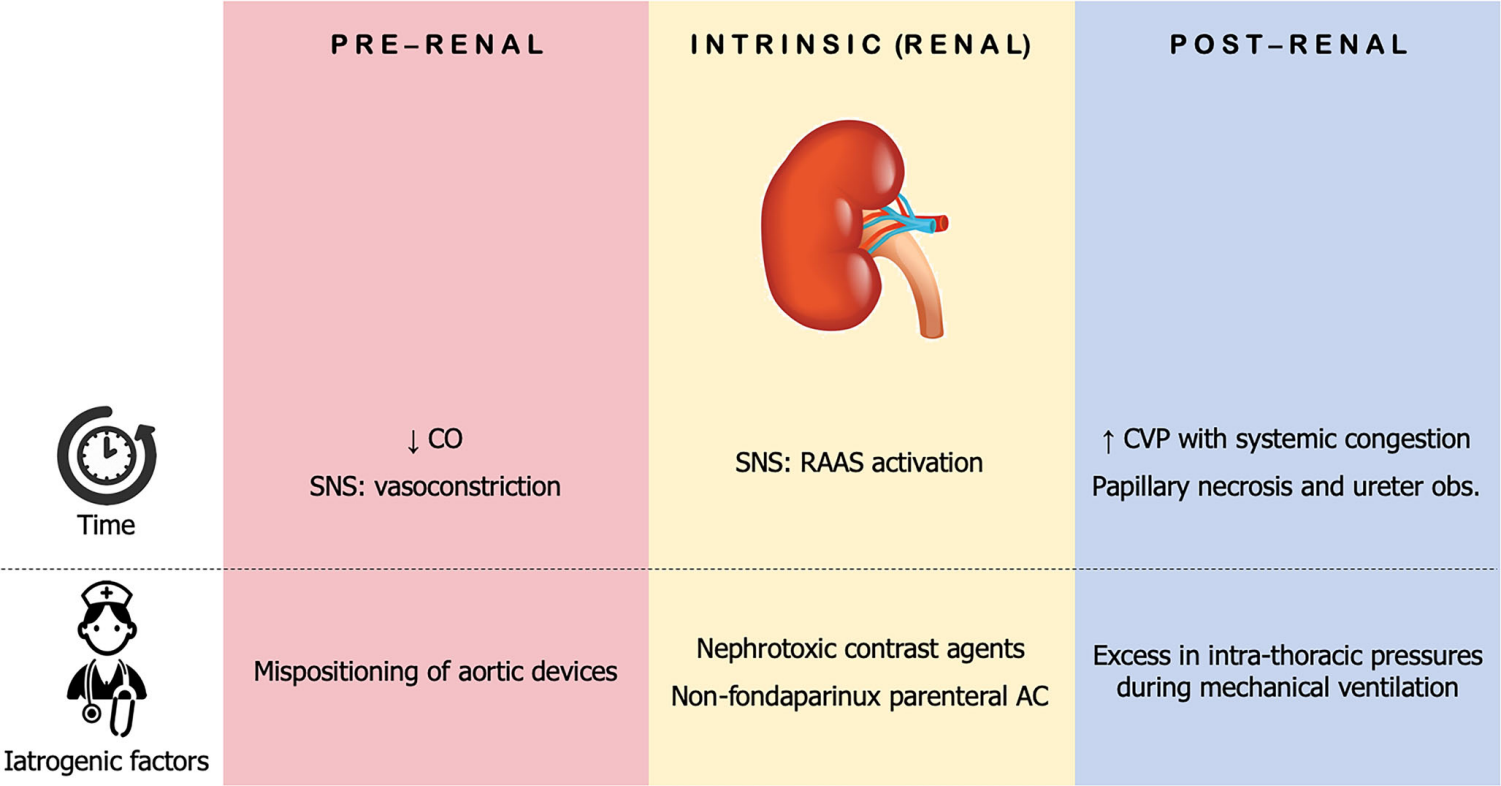
Prerenal, renal, and post-renal main causes of acute kidney injury complicating cardiogenic shock. Causes are distinguished as part of the natural history of the disease and as iatrogenic factors during in-hospital management. *AC*, anticoagulation; *CO*, cardiac output; *CVP*, central venous pressure; *obs*., obstruction; *RAAS*, renin-angiotensin-aldosterone system; *SNS*, sympathetic nervous system

Given this background, the interdependence between the heart and the kidney comes clear, with mutual detrimental amplification. The timing of occurrence of CS-AKI may vary among patients, with two main scenarios: those presenting with both CS and AKI at admission and those who develop AKI during hospitalization (Fig. 2). The latter cases may represent the natural evolution of the disease, as well as the complication of medical intervention. Iatrogenic factors indeed are often addressed as responsible or, at least, precipitating mechanism. For example, the use of nephrotoxic contrast agents as part of the investigation of CS complicating AMI may precipitate a preexistent borderline renal function alongside with ischemia-induced damages. Nevertheless, data from the Bremen STEMI registry did not find any correlation between the amount of the administered contrast agent and the onset of AKI, whilst the only driver was actually the impairment of the left ventricular (LV) function [25]. It is not clear whether the quantity of contrast could predict AKI in the specific subcategory of CS. Eventually, trans-femoral positioning of cardiac support devices (such as intra-aortic balloon pump) may complicate with renal artery occlusion, especially when delivered distally in the aorta [26].

## Clinical phenotypes

**Fig. 3:**
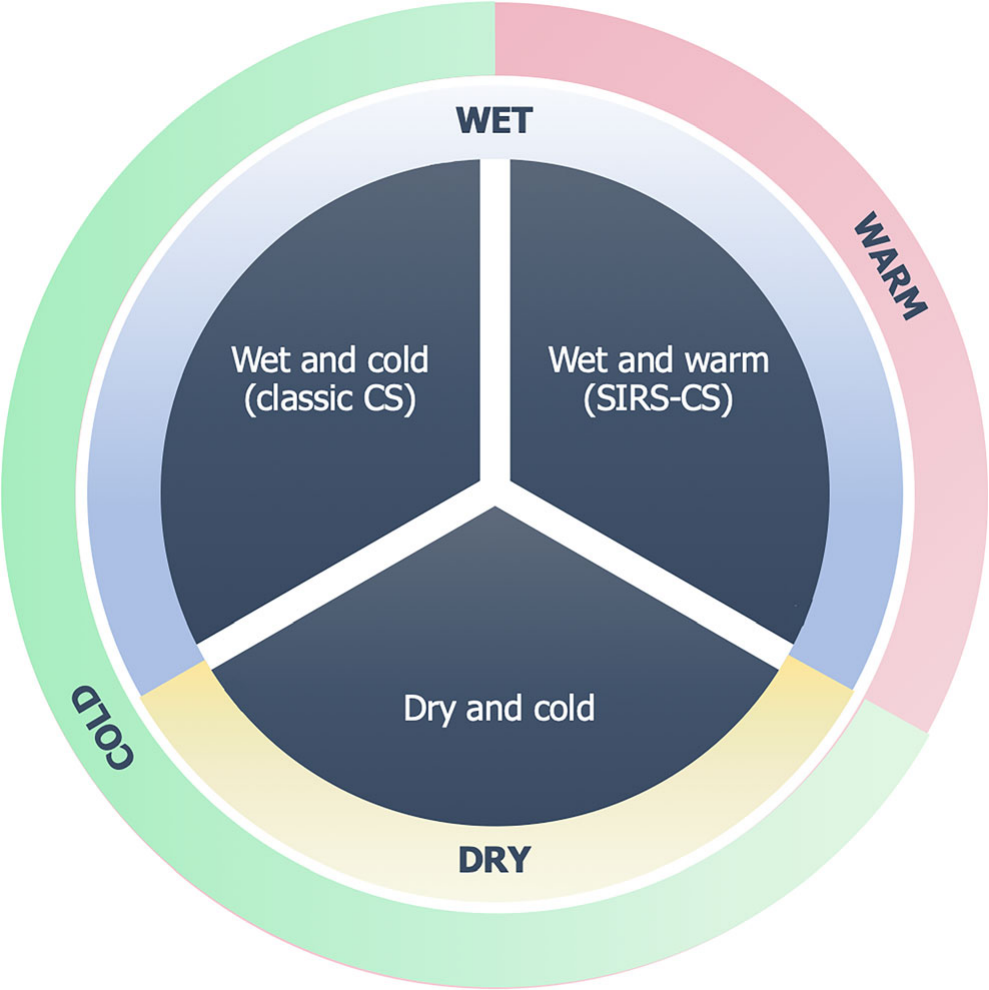
Clinical phenotypes of cardiogenic shock. Two additional types of cardiogenic shock have been described over the classic “wet and cold” phenotype. These are “dry and cold,” with cold extremities and no pulmonary congestion, and “wet and warm,” where a peripheral vasodilation is observed mainly as the consequence of a systemic inflammatory syndrome response. *CS*, cardiogenic shock; *SIRS*, systemic inflammatory response syndrome

CS is described as a status of hypoperfusion due to a significant reduction in cardiac index, leading to peripheral vasoconstriction and increased pulmonary capillary wedge pressure (PCWP) [27]. Although a reduced cardiac index is a necessary prerequisite for the diagnosis of CS, both peripheral vascular resistance and PCWP may vary among patients, as demonstrated by the SHould we emergently revascularize Occluded Coronaries for Cardiogenic shock (SHOCK) trial [2]. New phenotypes were then categorized, since a minority of patients could present as “wet and warm” or “dry and cold” over the traditional “wet and cold” (Fig. 3). With regard to the former cases, systemic inflammatory response syndrome was shown to occur also in the setting of cardiogenic shock, thus activating the inflammasome and leading to inappropriate systemic vasodilation. This can be due to two orders of factors: the release of cytokines—in particular TNF alpha—from the injured myocardium, and the release of endotoxins and bacteria translocating from the hypoxic intestine, thus leading to a catecholamine-unresponsive profile of shock [28].

The “dry and cold” phenotype includes patients with significant vasoconstriction but low or normal PCWP (6–12 mmHg). This is considered a diuretic-responsive class of patients who usually suffer from chronic heart failure and are less likely to have chronic kidney disease as a comorbidity [29]. Therefore, as clinical presentation may be heterogeneous, studies addressing potential differences in renal function and the likelihood of developing AKI are required for each class. As renal function can be affected in the settings of both peripheral and pulmonary congestion, it is reasonable to think that each phenotype may present with a various degree of renal impairment.

## Management

Whether therapy for CS is a tough challenge for clinicians when presenting alone, the combination of both CS and AKI is a further complication in the decision-making process. Indeed, most of the interventions are still made on an empirical basis rather than on solid results from clinical trials. A thorough invasive assessment and prompt interventions of both revascularization and replacement therapies are strongly recommended when needed.

### Diagnostic assessment

An in-depth assessment of hemodynamic parameters is key in the management of CS as well as in CS-AKI. CVP monitoring (normal values 3–8 mmHg) can predict the onset of AKI complicating CS, as it can reflect systemic venous congestion, with the kidneys that are no exception. CVP can be further influenced by the regulation of ventilatory parameters when mechanical ventilation is required (see “Ventilatory support”) [18].

Invasive arterial pressure measurement should also be encouraged. In particular, pulse pressure and stroke volume variations derived from arterial waveform demonstrated to prevent the onset of AKI in a setting of CS from post-resuscitated cardiac arrest [30]. More comprehensive studies are needed in order to assess whether this strategy may be successfully applied to CS from other causes.

A close monitoring of renal function and urine output is strongly recommended. The estimation of renal function can be achieved by the measurement of specific plasma proteins (such as creatinine and cystatin C) and dedicated biomarkers of renal injury, as well as by the monitoring of urine output. To our knowledge, only the study by Tarvasmäki et al. investigated renal function in the context of CS and performed a between-methods comparison using KDIGO criteria for AKI [31]. AKI according to cystatin C was defined on a par with creatinine. The elevation of both molecules did not show a significant difference in the prediction of outcomes; thus, their use for AKI stratification is comparable and valuable. On the contrary, KDIGO cut-off of 0.5 mL/kg/min was not that advantageous, albeit a stricter cut-off of 0.3 mL/kg/min proved effective and independently associated with 90-day mortality. The introduction of renal injury biomarkers (i.e., neutrophil gelatinase–associated lipocalin [NGAL]) in clinical practice may play a useful role, as their increase in plasma occurs earlier than the changes in parameters of function and is less dependent on hemodynamic modifications [32] (see Table 2). Although they did not provide any additional prognostic information, predicting AKI before it actually occurs may influence therapeutic decisions, as a prompter intervention with mechanical circulatory support devices may avoid its onset and therefore a worsening in outcome [33].

**Table 2:** Diagnostic work-up and management in patients with CS-AKI, according to invasive and non-invasive monitoring strategies and laboratoristic and echocardiographic findings. Name of the parameters, normal values, frequency of measurements, and further comments are here reported. *AKI*, acute kidney injury; *CS*, cardiogenic shock; *KDIGO*, Kidney Disease: Improving Global Outcome; *NGAL*, neutrophil gelatinase–associated lipocalin and kidney injury molecule; *SBP*, systolic blood pressure

Parameter	Normal values	Frequency	Comments and management implications
Invasive and non-invasive monitoring			
Arterial invasive blood pressure monitoring	SBP ≥ 90 mmHg	Continuous	Affords tissue perfusion and prevents peripheral vasoconstriction, thus reducing cardiac afterload. Pulse pressure and stroke volume as derived by arterial waveform predict AKI in CS after resuscitated cardiac arrest
Heart rate	60–100 bpm	Continuous	High values increase heart oxygen consumption
Central venous pressure	3–8 mmHg	Continuous	Reflects the venous return to the right heart from periphery, as well as the ability of the heart to pump into the arterial tree. Its increase is associated with a higher incidence of AKI and may guide right ventricular-focused assessment
Arterial oxygen saturation	≥ 94%	Continuous	Estimates the content of oxygen in arterial blood. Its information varies depending on the sampling point, whether in great vessels or in capillaries
Central venous oxygen saturation	≥ 70%	Continuous/every 4–6 h	Estimates the balance between oxygen delivery and consumption, thus reflecting tissue extraction of oxygen in relation to heart pump function
Respiratory rate	12–20 breaths per minute	Every 8 h	It is often controlled by the clinician because of the need for mechanical invasive support
Urine output	0.5 mL/kg/h	Hourly	A rough but effective marker of renal function. Urinary catheterization has to be performed in every patient. Whether a KDIGO-stated cut-off of 0.5 is generally accepted, a stricter one of 0.3 is more related to 90-day mortality in the setting of CS-AKI
Laboratory findings			
Lactates	0.5–1.6 mmol/L	Every 4–6 h	Represents a marker of end-organ hypoperfusion, as it indicates a shift to anaerobic metabolism. Sample-to-sample differences in lactate values are more sensitive of the clinical outcome than single values
Serum creatinine	0.8–1.3 mg/dL (men) 0.6–1.1 mg/dL (women)	Every 12–24 h	A marker for the estimation of renal function. Completely filtered, partially secreted in the proximal tubule. Its elevation is significantly delayed with respect to the renal damage
Serum cystatin C	0.60–1.55 mg/L	First phases (if available)	A marker for the estimation of renal function. Completely filtered, no secreted or reabsorbed. Less dependent on age, gender, ethnicity, and muscle mass compared with creatinine. Its elevation is significantly delayed with respect to the renal damage
NGAL	28.7–167.0 ng/mL	First phases (if available)	A marker of renal damage. Its increase is way more precocious than markers of function, hence raising awareness of renal involvement
Echocardiographic findings			
Stroke volume	50–80 mL	Daily	Evaluates left ventricular function, even if it is strictly dependent on preload and afterload. It affords a between-days comparison in pump function
Left ventricular ejection fraction	55–60%	Daily	Evaluates left ventricular function. Attention has to be paid to any pathological condition that falsely overestimates the ejection fraction, i.e., severe mitral regurgitation secondary to LV dilatation or papillary dysfunction or ischemic septal ventricular defect
E mitral wave deceleration time	> 150 ms	First phases	As part of the assessment of diastolic function, together with E/A (restrictive pattern if values ≥ 2). Values below the reference limit represent a strong predictor of outcome in the acute phase
Right ventricular fractional area change (RV-FAC)	≥ 35%	First contact and anytime right heart involvement is suspected	Evaluates right ventricular function. Attention has to be paid to a pseudo-normalization of this value under conditions of volume overload
Right ventricular free wall longitudinal strain	< − 13.1%	At admission and at 48 h	Evaluates right ventricular performance with higher sensitivity and reproducibility than RV-FAC. Useful in the prediction of right ventricular failure after left ventricular assisted device implantation
Hepatic veins flow	−	First contact and anytime right heart involvement is suspected	Evaluates right ventricular function. A bi- or tri-phasic waveform with D-wave greater than S-wave may suggest right heart failure, as well as tricuspid regurgitation. An irregular pattern of the waveform may suggest arrhythmias

### Cardiogenic shock related to ischemic heart disease

As CS represents the ultimate step for multiple severe injuries to the myocardium, defining and readily solving the underlying cause should be considered the gold standard of treatment. Since AMI is still the leading etiology for CS, coronary angiography with a view to revascularization is often the treatment of choice. By its side, percutaneous revascularization includes the utilization of contrast agents that are nephrotoxic, so possibly triggering AKI, even if the correlation between AKI and the amount of contrast has been questioned [25]. Furthermore, when prompt and invasive evaluation of the coronary anatomy is required, unfractionated heparin should be considered the anticoagulant treatment of choice, given that both low molecular weight heparin and fondaparinux may precipitate AKI [1, 34]. A shared guideline is missing with regard to the strategy of revascularization in the context of multivessel disease, since current studies came to different conclusions. Results from the CULPRIT-SHOCK trial demonstrated an increase in mortality or need for RRT for patients receiving an “all-in-one” revascularization, thus preferring a culprit-dedicated approach [35]. Accordingly, the treatment of non-culprit artery disease should be postponed to a time when clinical stabilization is reached. A recent subanalysis from the same trial also showed that no different approaches should be undertaken between men and women, even if the latter presented with a different profile of risk [36].

### Ventilatory support

When a ventilatory support is required, a setting with low tidal volumes is strongly recommended in order to reduce the incidence of RV failure and to achieve an appropriate venous return, thus avoiding venous renal stasis and edema. [37]. Additionally, despite initial concerns regarding a worsening in cardiac output, moderate values of positive end-expiration pressure (PEEP, 5 cmH_2_O) were able to reduce LV oxygen demand as well as to improve myocardial oxygen delivery, perhaps due to reduced afterload and preload, with consequent LV unloading [38–41]. Indeed, patients experiencing CS are more prone to be afterload-dependent rather than preload-dependent, with the exception of RV failure and/or hypovolemia. In these two scenarios, clinicians should initiate a low PEEP regimen (3–5 cmH_2_O) only when euvolemic state is achieved, with a view to up-titration. Close evaluations of blood gas analysis can help to adjust the ventilatory strategy, as well as to evaluate lactates, whose high levels were found to predict persistent AKI [42].

### Pump failure and mechanical circulatory support

LV pump failure, as assessed by several hemodynamic (i.e., cardiac index), laboratoristic (lactates) and non-invasive parameters (LV ejection fraction, diastolic function, RV performance), requires adequate support to antagonize peripheral hypoperfusion. A long-standing experience with inotropes has been collected, reporting controversial findings. Norepinephrine and dobutamine are the first-line agents for patients with evidence of CS, but their use should be as limited as possible given the increase in arrhythmic risk. Moreover, the higher the dose of catecholamines required, the higher is the likelihood of developing persistent AKI [42]. Novel agents have been tested in order to overcome the high burden of adverse effects from first-generation drugs. Among them, the infusion of levosimendan on top of ineffective treatment with catecholamines was well tolerated and responsible for improved cardiovascular hemodynamics, as estimated by cardiac power and cardiac power index. Results were further confirmed also for patients requiring RRT. Hypotension was not significantly observed even if levosimendan holds an “inodilator” role [43]. Despite these promising observations, no benefits were observed regarding mortality [44].

In the era of replacement therapies, a breakthrough in CS and CS-AKI management was expected. Unfortunately, most of the studies demonstrated a poor contribution of first-generation devices supporting cardiac function. Routine use of intra-aortic balloon pump (IABP) is contraindicated in the management of CS [45, 46]. Data from new-generation devices are quite scarce, but Impella showed no superiority to IABP itself in mortality, at the cost of a higher bleeding risk [47]. Only a very early use of Impella—also before PCI was performed—showed improvements in patient outcome, according to analyses of the USpella registry [48]. This affords a proper LV unloading, perhaps accelerating muscle recovery when combined with revascularization of the culprit artery. Although data regarding benefits in the prevention or amelioration of AKI are incomplete, it is known by previous findings that CS-AKI patients are more likely to require temporary mechanical support devices (MCS) than those without AKI [9]. Given their increased need for MCS, early implantation may be crucial in the management of AKI before it occurs, with a likely improvement in outcome.

The use of the extracorporeal membrane oxygenation (ECMO) has increased in the setting of CS, with the veno-arterial being the most indicated technique, especially when both cardiac and respiratory failures are documented. The rationale of its use includes both a supply in the oxygenation of blood and a supplemental pump function that is complementary to the one from the failing heart, thus improving hemodynamics. Concerns were raised about a possible increase in afterload, requiring a further unloading device or intervention in combination (central ECMO or LV apical vent) [49]. The use of veno-arterial ECMO showed transient benefits in the context of post-cardiotomy CS, even if AKI may be a complication itself of this technique, ranging from 70 to 85% depending on centers and requiring RRT in about half of patients [50]. The onset of AKI during ECMO may be caused by infections, hypotension, an inflammatory-like reaction to the extracorporeal support and fluid overload [51, 52]. Moreover, the longer is the time of support, the higher is the risk for major complications, including severe bleedings (especially intracranial hemorrhages), hemolysis, infections, and multiorgan failure [53]. Of note, complications may be at least partially attributed to the anticoagulant regimen more than to the ECMO itself, but are still unacceptably high [54]. Trials assessing the actual utility of ECMO in CS and CS-AKI are needed, especially in the widespread context of AMI.

### Renal replacement therapy

The use of continuous veno-venous hemofiltration (CVVH) as a technique of RRT was tested in the setting of post-cardiotomy CS-AKI. When promptly used (especially in the early perioperative period), CVVH at high rates was associated with better in-hospital and long-term survival [54]. This finding is in agreement with the superiority of CVVH to intermittent hemodialysis, whose fluid shifts are scarcely tolerated in patients with CS. However, the impact of RRT on outcome decreased when the number of comorbidities increased; hence, a multifocused, optimized treatment for each clinical condition should be perceived [10]. The timing of RRT is widely debated, but it should be considered at stage 2 kidney injury or whenever life-threatening changes in fluid, electrolyte, and acid-base balance precipitate the need for dialysis [4].

## Prognosis

Despite recent advances, especially with regard to replacement therapies, CS is still a life-threatening condition with a high mortality rate. Renal involvement should be considered more the rule than the exception, as kidneys receive 20–25% of the whole blood supply. The superimposition of AKI is a deleterious clinical complication that further affects prognosis in a direct proportional fashion to the grade of renal damage. Higher mortality rates, length of stay, and costs were observed in patients requiring replacement therapy as compared with those who did not [9]. Albeit veno-venous hemofiltration actually improved outcomes, RRT following AMI-related CS was also found to predict long-term risk of chronic dialysis and mortality [11].

Since the onset of AKI heavily affects the outcome, a switch in the focus from renal function to renal damage should be strongly promoted. This may afford an earlier detection of renal impairment, guiding the clinician to more precocious invasive strategies that prevent AKI to occur or to worsen. In these terms, the introduction in clinical practice of biomarkers of renal injury may be a step forward a better comprehension and treatment of CS-AKI, as their increase in plasma is more rapid than markers of function. Further studies should assess whether device implantation guided by these biomarkers may actually improve outcome.

## Conclusions

Despite the large number of scenarios in which CS and AKI can coexist, just the one regarding AKI complicating AMI-related CS is well described, hence representing an important gap in evidence. CS-AKI holds high complexity and high fatality rate, and growing evidences should discourage clinicians to consider it as a unique syndrome. Whether the abrupt depression in cardiac function is the common thread through the entire disease spectrum, variability in peripheral and pulmonary vascular tone has been observed. This complicates the understanding of the disease, but on the other hand opens the possibility to tailored clinical trials and therapies that rely on the combination of hemodynamic parameters. A thorough neurohormonal investigation may further integrate this approach and should be collected from each patient.

As only a very early use of MCS showed beneficial, it may follow that a precocious LV unloading affords the muscle to recover, even before proper revascularization is restored. The optimal window in order to improve prognosis appears to be really short in time, thus requiring a well-organized connection between the territory, spoke, and hub centers. Patient’s and emergency medical system’s delays should be as reduced as possible, with no hesitation in recurring to MCS.

Clinical trials for CS-AKI are still poor, despite its relatively high frequency, the high lethality rate, and the amount of unanswered questions. Although the emergency setting of care often complicates the design and the feasibility of clinical trials, many efforts should be made to obtain a better comprehension of the disease, especially with concerns to the correct timing of interventions other than revascularization.

## Abbreviations

AKI: Acute kidney injury
AMI: Acute myocardial infarction
CRS: Cardiorenal syndrome
CS: Cardiogenic shock
CVP: Central venous pressure
CVVH: Continuous veno-venous hemofiltration
ECMO: Extracorporeal membrane oxygenation
IABP: Intra-aortic balloon pump
LV: Left ventricular
MCS: Mechanical support devices
RV: Right ventricular
PCWP: Pulmonary capillary wedge pressure
PEEP: Positive end-expiration pressure
RRT: Renal replacement therapy
